# A broad analysis of resistance development in the malaria parasite

**DOI:** 10.1038/ncomms11901

**Published:** 2016-06-15

**Authors:** Victoria C. Corey, Amanda K. Lukens, Eva S. Istvan, Marcus C. S. Lee, Virginia Franco, Pamela Magistrado, Olivia Coburn-Flynn, Tomoyo Sakata-Kato, Olivia Fuchs, Nina F. Gnädig, Greg Goldgof, Maria Linares, Maria G. Gomez-Lorenzo, Cristina De Cózar, Maria Jose Lafuente-Monasterio, Sara Prats, Stephan Meister, Olga Tanaseichuk, Melanie Wree, Yingyao Zhou, Paul A. Willis, Francisco-Javier Gamo, Daniel E. Goldberg, David A. Fidock, Dyann F. Wirth, Elizabeth A. Winzeler

**Affiliations:** 1 Department of Pediatrics, School of Medicine, University of California San Diego, 9500 Gilman Drive 0741, La Jolla, California 92093, USA; 2 Department of Immunology and Infectious Disease, Harvard T.H. Chan School of Public Health, 665 Huntington Avenue, Boston, Massachusetts 02115, USA; 3 Infectious Disease Program, The Broad Institute, 415 Main Street, Cambridge, Massachusetts 02142, USA; 4 Department of Medicine and Microbiology, Washington University School of Medicine, St Louis, Missouri 63110, USA; 5 Department of Microbiology and Immunology, Columbia University College of Physicians and Surgeons, New York, New York 10032, USA; 6 Tres Cantos Medicines Development Campus, Malaria DPU, GlaxoSmithKline, Severo Ochoa 2, Tres Cantos, 28760 Madrid, Spain; 7 The Genomics Institute of the Novartis Research Foundation, 10675 John J Hopkins Drive, San Diego, California 92121, USA; 8 Medicines for Malaria Venture, PO Box 1826, 20 route de Pre-Bois, 1215 Geneva 15, Switzerland

## Abstract

Microbial resistance to chemotherapy has caused countless deaths where malaria is endemic. Chemotherapy may fail either due to pre-existing resistance or evolution of drug-resistant parasites. Here we use a diverse set of antimalarial compounds to investigate the acquisition of drug resistance and the degree of cross-resistance against common resistance alleles. We assess cross-resistance using a set of 15 parasite lines carrying resistance-conferring alleles in *pfatp4*, cytochrome *bc*_*1*_, *pfcarl*, *pfdhod, pfcrt*, *pfmdr, pfdhfr,* cytoplasmic prolyl t-RNA synthetase or *hsp90*. Subsequently, we assess whether resistant parasites can be obtained after several rounds of drug selection. Twenty-three of the 48 *in vitro* selections result in resistant parasites, with time to resistance onset ranging from 15 to 300 days. Our data indicate that pre-existing resistance may not be a major hurdle for novel-target antimalarial candidates, and focusing our attention on fast-killing compounds may result in a slower onset of clinical resistance.

## Abstract (web summary)

It is unclear whether new antimalarial compounds may rapidly lose effectiveness in the field because of parasite resistance. Here, Corey *et al.* investigate the acquisition of drug resistance and the extent to which common resistance mechanisms decrease susceptibility to a diverse set of 50 antimalarial compounds.

## Graphical abstract

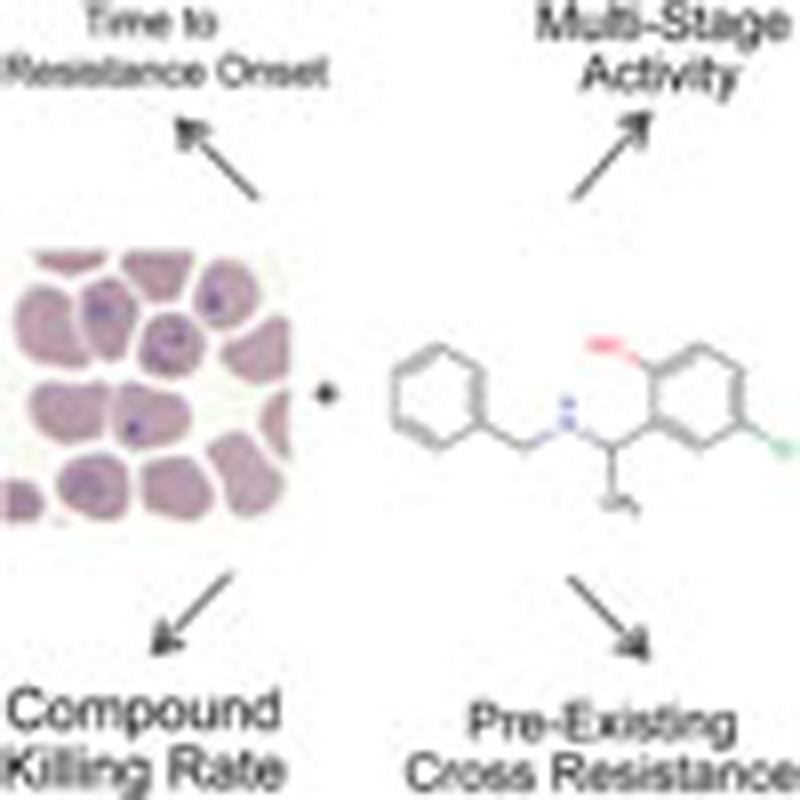

Malaria remains a devastating disease, with an estimated 198 million cases (uncertainty range 124–283 million) and 584,000 deaths (uncertainty range 367,000–755,000) in 2013 alone, affecting primarily children under the age of five^1^. Given that the current vaccine available provides only moderate protection^2^, chemotherapeutics constitute the best clinical tools available for the prevention and treatment of malaria. Currently, A number of effective drug therapies exist, most of which target the malaria parasite during its replicative cycle within human erythrocytes, the lifecycle stage that is associated with clinical manifestations of malaria^3^. These consist of 4-aminoquinolines including chloroquine, piperaquine and related compounds; antifolates such as pyrimethamine and cycloguanil; alkanolamines such as halofantrine and lumefantrine; endoperoxides such as artesunate, artemisinin and artemether; and newer synthetic compounds. Antimalarial treatments are typically administered as combination therapies, and artemisinin-based combination therapies (ACTs, such as artemether-lumefantrine) currently represent the therapy class that is most effective and that is the standard of care recommended by the World Health Organization (WHO).

While ACTs have been a highly effective frontline therapy, particularly against multidrug-resistant *Plasmodium falciparum* infections, there is now evidence of resistance emerging to artemisinin and its derivatives^4^,^5^,^6^,^7^. Clinical trials with artemisinin mono-therapies have shown that these compounds are taking considerably longer to clear malaria infections in Southeast Asia—typically twice as long as observed a decade ago. Given that parasites have already acquired resistance to several partner drugs, treatment failures are beginning to be observed with combination therapies^8^,^9^. Thus, new compound classes, ideally with new mechanisms of action, are urgently needed if the gains of the last decade are to be sustained.

In anticipation of eventual widespread ACT failure, there has been a focused and coordinated effort to place new antimalarial drug candidates into the drug development pipeline (see http://www.mmv.org/research-development/rd-portfolio). Leads from phenotypic screens in particular are being progressed into molecules that are suitable for testing in clinical trials. An open question, however, is whether small molecules from phenotypic screens will lead to the identification of new druggable targets and pathways that do not rapidly lose effectiveness in the field because of acquired and pre-existing parasite resistance.

Here we use a set of 50 antimalarial compounds identified in phenotypic screens^10^,^11^,^12^,^13^ to systematically evaluate whether resistant parasites can be selected and whether or not pre-existing resistance mechanisms confer resistance using a panel of strains containing mutations in a variety of genes, including *P. falciparum* cyclic amine resistance locus (*pfcarl)*^14^, cytochrome *bc1* (refs 15, 16, 17) and *P. falciparum* ATPase 4 (*pfatp4*)^18^,^19^,^20^. Here we show that pre-existing resistance is less likely to be a problem but that the *de novo* acquisition of resistance occurs rapidly for many compounds. We highlight a set of antimalarial compounds that have thus far defied attempts to create drug-resistant parasites in a variety of different laboratories and identify features that are shared by all, including a rapid rate of killing and lack of pre-existing resistance.

## Results

### Initial selection of compounds

To systematically investigate both the *de novo* acquisition and pre-existing landscape of drug resistance, we assembled a set of 50 diverse compounds selected from *P. falciparum* asexual phenotypic screens^10^,^11^,^12^,^13^. Compounds were initially chosen based on potency, demonstrated by activity against asexual blood stages ranging from 23 nM to 1.67 μM EC_50_ with most compounds having an EC_50_ of <1 μM in the *P. falciparum* strain 3D7 as measured by a hypoxanthine incorporation assay. To minimize non-novel pharmacophores, compounds were compared against the scaffolds of clinical antimalarials, eliminating candidates with similar structures.

**Figure 1:**
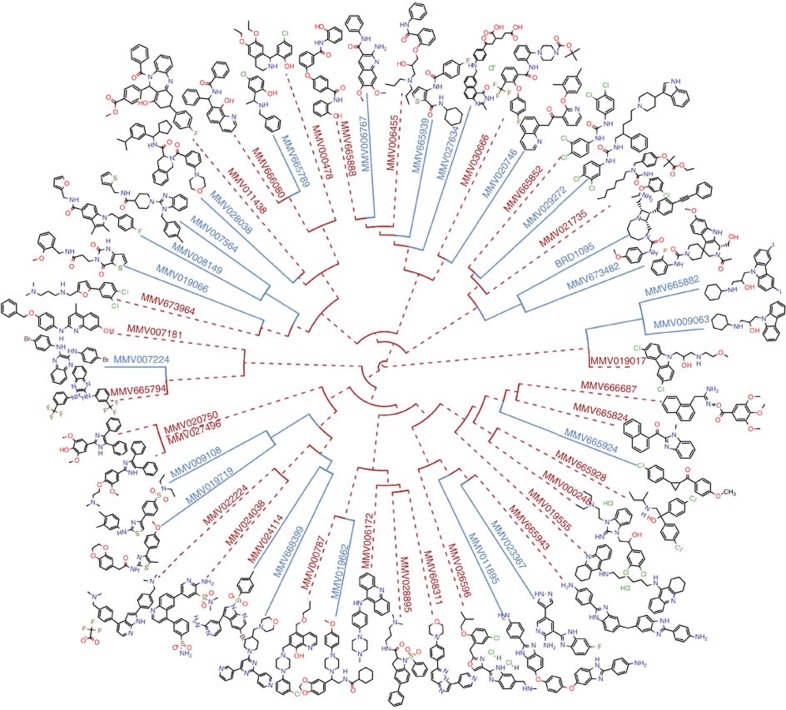
Chemically diverse compound set. Hierarchical clustering of the 50 compound set. Compounds were clustered by a maximum substructure similarity Tanimoto coefficient. *In vitro* selections that were successful in yielding resistant parasites are highlighted in blue, whereas compounds where resistance development was unsuccessful are highlighted in dashed red.

Compounds in our set ranged in molecular weight from 261 to 574 g·mol^−1^, with 42 compounds having drug-like properties (compliant with Lipinski's rule of five) and the remaining 8 identified as probe-like compounds. We sought to maximize chemical diversity in our set by eliminating similar compounds as indicated by the Tanimoto coefficient, since compounds with Tanimoto coefficients >0.85, a quantitative measurement of chemical scaffold similarity, are thought to have similar biological activity to one another^21^,^22^. The resulting compound set displayed an average Tanimoto coefficient of 0.186, ranging from 0.093 to 0.923 (Fig. 1). Although a few compounds were similar to one another (particularly two carbazoles: MMV009063 and MMV665882) a majority of the set was diverse, possessing a variety of functional groups and heterocyclic substructures.

### Compound evaluation against a panel of resistant clones

Multidrug resistance alleles, including mutations in *P. falciparum* chloroquine resistance transporter (*pfcrt)* and *P. falciparum* multi-drug resistance transporter 1 (*pfmdr1*) are very common in field isolates. Therefore, we sought to assess the degree to which pre-existing resistance alleles would contribute to a loss of potency for this diverse collection of small molecule compounds. Fifteen clones derived from three main parent lines were chosen to maximize diversity in mutated pathways and genetic backgrounds, thus representing the variety of resistance seen in the field: 3D7 (ref. 23), W2 (ref. 24) or Dd2 (ref. 25). The 3D7 line originates from the clone of a Netherlands clinical isolate strain^23^,^26^ and is generally considered to be drug sensitive, though it does convey resistance to sulfadoxine. Conversely, W2 (ref. 24, 27) and Dd2 (ref. 25) are multi-drug-resistant lines originating from the Indochina III/CDC isolate, which contain point mutations in *pfcrt* as well as amplifications in *pfmdr1* and GTP cyclohydrolase^27^.

A total of nine known drug-resistance genes were represented by our strain set, containing validated critical single nucleotide variants (SNVs) or copy number variants as well as a handful of additional background mutations. One strain, TM90C2A, was a clinical isolate from Thailand^28^, while the remaining 15 lines were created through *in vitro* evolution. Clones contained one or more resistance-conferring alleles in the folate pathway^28^, *pfcrt* (ref. 29), *P. falciparum* dihydroorotate dehydrogenase (*pfdhodh*)^30^, *pfcarl* (ref. 14), prolyl t-RNA synthetase^31^ or heat shock protein 90 (*hsp90*). We also investigated multiple cytochrome *bc*_*1*_ (refs 15, 16, 17) and *pfatp4* (refs 18, 19, 20) alleles, including three alleles in the cytochrome *bc*_*1*_ Q_o_ site, which confer resistance to atovaquone^15^ or a tetracyclic benzothiazepine^16^ and one allele in the cytochrome *bc*_*1*_ Q_i_ site conferring resistance to a benzylsulfonamide^17^. The five PfATP4 mutant lines possess alleles that map to the transmembrane channel of the sodium-dependent ATP4 transporter, and were acquired by exposing parasites to sub-lethal concentrations of a spiroindolone^18^, an aminopyrazole^19^, or other scaffolds from the Tres Cantos Antimalarial Set library^20^. These two resistance genes were represented by multiple strains since critical SNV mutations may be located within an active site or result in a change in membrane potential, resulting in each mutation only affecting a subset of PfATP4 or cytochrome *bc1* inhibitors.

To evaluate potential overlapping activity against known antimalarial drug targets, we performed dose-response studies with each compound against our assembled set of drug-resistant lines and their corresponding parents, in an asexual blood stage proliferation assay (Table 1). Comparisons were made to the drug-sensitive strain 3D7 when a parent was unavailable (TM90C2A, W2 and Dd2). As laboratories conducted EC_50_ assays with different strains and protocols, all comparisons were made between assays from the same laboratory and under the same assay conditions. Compounds displaying >5-fold EC_50_ shifts relative to the parent or sensitive strain were flagged as having potentially non-novel targets. Average fold shifts per strain ranged from 0.82 to 2.34, with a median of 1.23 indicating there was not common resistance to any one resistant clone. No resistance patterns were observed when classifying the compounds based on chemical structure, but this was not surprising given the diversity of compounds chosen. On the other hand, given that these confirmed compounds were derived from larger unconfirmed hit lists to which some filtration criteria may have been applied (for example, elimination of obvious dihydrofolate reductase inhibitors^32^), some^10^,^11^,^12^,^13^ pre-existing bias may exist in the set.

**Figure 2:**
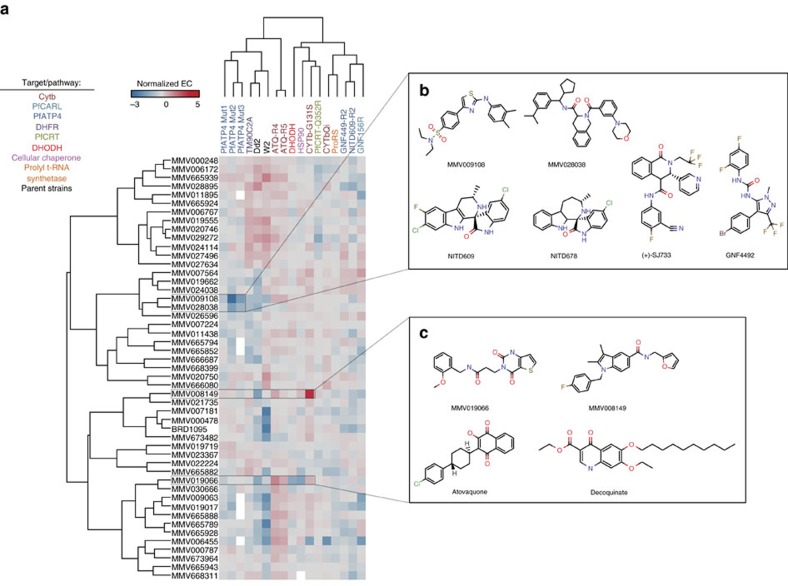
Cross-resistance fold shifts observed in compound set. (**a**) A total of 15 resistant strains were tested with each MMV compound to identify potential pre-existing cross-resistance. Calculating the fold shifts between each clone and either a corresponding parent or a drug sensitive 3D7 strain generated the heatmap. To normalize conferred resistance and sensitivity, the natural log of each fold shift is displayed. Fold shifts instead of raw data were used as multiple assays were run with varying times and detection indicators. Incomplete cross-resistance assays are depicted in white. All assays were run in triplicate. For one compound (BRD1095), a close analogue (BRD3444) was analysed for TM90C2A and PfATP4-Mut1–3. SMILEs for all compounds are listed in Supplementary Data 1. (**b**) Chemical structures of the two MMV compounds (MMV009108 and MMV028038) with increased efficacy against one or more *pfatp4* mutated clones. Both compounds displayed low structural similarity to a number of other known *pfatp4* inhibitors. (**c**) Chemical structures of the two MMV compounds (MMV019066 and MMV008149) with decreased efficacy against one or more cytochrome *bc1* mutated clones. Atovaquone and decoquinate, two other cytochrome *bc1* inhibitors, were structurally significantly different.

Alleles in the strain set generally did not confer resistance to the vast majority of the compounds (Fig. 2a), with only two compounds (MMV019066 and MMV008149) losing efficacy in some resistant lines relative to their parent clones (EC_50_ fold-shifts >5-fold). ATQ-R4, bearing a Q_o_ cytochrome *bc*_*1*_ allele, was resistant to a propanamide, MMV019066, (*P* value=0.0016) with an EC_50_ fold-shift of 16 × , as determined by a one-way analysis of variance (ANOVA) analysis with a Dunnett's multiple comparison correction. In addition, CYTb-G131S, a cytochrome *bc*_*1*_ Q_o_ allele, demonstrated complete resistance to MMV008149, a carboxamide, with a >38-fold change in EC_50_. Statistically significant resistance was seen against MMV019066 and MMV008149 in other cytochrome *bc*_*1*_ Q_o_ mutants (ATQ-R5 and ATQ-R4, respectively), but the EC_50_ fold-shifts seen were under the threshold value set for the study. Neither compound shared any structural features with other cytochrome *bc*_*1*_ inhibitors, including atovaquone and decoquinate (Fig. 2b).

As had been previously observed^19^, mutations in PfATP4 resulted in parasites that were more sensitive to unrelated compounds: ATP4-Mut2 exhibited sensitivity to a sulfonamide (MMV009108—*P* value=0.0001; 23 × fold-shift) and a carboxamide (MMV028038—*P* value=0.0007; 5 × fold-shift). ATP4-Mut3 additionally conferred sensitivity to MMV009108 (*P* value=0.0047; 5.2 × fold-shift). Neither compound was structurally similar to spiroindolones, pyrazoles or dihydroisoquinolones, all previously identified PfATP4 inhibitors (NITD609 (ref. 18), KAE678 (ref. 18), GNF4492 (ref. 19) and (+)-SJ733 (ref. 20)), with a Tanimoto coefficient range of 0.127–0.34 (Fig. 2c).

Finally, two compounds (MMV665939 and MMV028895) lost efficacy in the parent strains W2 or Dd2 when compared with the 3D7 sensitive strain within the same lab. MMV665939 demonstrated fold shifts in W2 compared with 3D7 (EC_50_ fold-shift—9.5 ×), as well as in the other two W2-based clones (PfATP4-Mut1: 16.6 × , PfATP4-Mut2: 18.9 ×) compared with 3D7. Fold shifts in Dd2 compared with 3D7 were also seen in MMV665939, though these shifts were less severe (1.5–3.1 × ; average 2.4 ×). MMV028895 showed reduced efficacy in Dd2 (EC_50_ fold-shift 7.1 ×), which was also seen throughout the other Dd2 clones (2.0–7.5 × ; average—5.5 ×), but unlike MMV665939 maintained potency against W2 strains. These efficacy changes in Dd2 and W2 are most likely due to *pfmdr*, given that W2 and Dd2 contain additional copies of the multi-drug resistance gene (2 and 3–4 copies, respectively) when compared to 3D7.

### Selection of resistant parasites

While we found an overall lack of pre-existing resistance, analysing the onset of resistance was critical, as compounds resulting in rapid resistance are not ideal for clinical development. We therefore sought to create drug-resistant parasites using a variety of different selection methods. We implemented either a high-dose method or a ramp-up/pulse method for our *in vitro* selections. Two of the 50 compounds—MMV028895 and MMV665824—were removed from the selection study during experimentation. MMV665824 was removed due to a significant loss of potency, while MMV028895 exhibited cross-resistance to resistant parasites generated by MMV007564. Out of the remaining 48 compounds, resistant parasites were obtained for 23 (Supplementary Data 1). As each selection was performed in triplicate with three independent cultures, we succeeded in generating a total of 66 resistant cultures. Parasites resistant to MMV026596 were not obtained despite 100 days of selection, but the cultures acquired hypersensitivity to mefloquine with a 10-fold reduction in EC_50_ compared with its parental line, 3D7. For the remaining 24 compounds, acquisition of resistant parasites was unsuccessful despite numerous attempts over an extensive period of time.

To determine if the cross-resistance assay was predictive, we sought to determine the target of MMV008149, the compound resulting in the largest EC_50_ fold-shift (>38 ×) within the cross-resistance assay set by fully sequencing six parasite clones that had acquired resistance to MMV008149. The EC_50_ values for the resistant lines ranged from 1.67 μM to 10.06 μM, a 3–21 × EC_50_-fold change when compared with the Dd2 parent (EC_50_=485 nM).

Genomic DNA (gDNA) was isolated from the Dd2 parent and six clones (two clones per resistant selection flask), and samples were prepared for whole-genome sequencing. Samples were sequenced to >60 × coverage using paired end reads and aligned to the 3D7 reference genome and variants were called with HaplotypeCaller (GATK). Comparing the nucleotide variation found in the resistant samples to the Dd2 parent clone, which had been isolated immediately before selections, we identified genomic changes that had presumably occurred during selection. Following this comparison, 19 mutations were identified: 11 SNVs and eight insertion/deletions (INDELs) (Table 2, Supplementary Data 2). INDEL mutations were comprised of intergenic (three), codon INDEL (three), intronic (one) and frame-shift (one) mutations. The SNV set showed a mixture of intergenic (five), synonymous (three) and non-synonymous (three) mutations. Comparing all six clones, one gene was mutated across all samples: cytochrome *bc1*. Additionally, variant positions correlated well to EC_50_ fold changes, with the lower fold changes (3.4–5.9 ×) corresponding to the G131S amino acid change, and the higher EC_50_ fold-shifts (18–21 ×) corresponding to the Y126C and V284L amino acid changes. Two of the mutations, G131S and Y126C, were contained in the Q_o_ site, while the third mutation (V284L) was not in either the Q_o_ or Q_i_ binding region^29^. G131S was also the major mutation found in CYTb-G131S, the cross-resistant strain predicting cytochrome *bc1* as a potential target for MMV008149. These results support that the cross-resistance assay was able to successfully identify compounds with overlapping targets, and made us more confident that our compound set consisted of novel targets.

### Multi-stage activity profiling

As drugs that eliminate multiple stages of the parasite lifecycle will be critical components in global efforts to eliminate malaria^10^, we were interested in knowing whether the compounds in our study had additional activities against either the liver and/or transmission stages of the parasite lifecycle. To determine this, we first evaluated the compounds in additional phenotypic assays. The first assay was a *P. berghei* hepatocyte invasion and development assay, which predicts causal prophylactic activity. Hepatocyte toxicity was additionally tested to identify false-positive activity in the liver stage due to host-cell toxicity. Assays were executed in duplicate using a 12-point EC_50_ curve at starting concentrations of 5 μM or 50 μM (Supplementary Table 1). Positive and negative controls testing for hepatocyte toxicity and liver stage activity included atovaquone (PfLuc EC_50_:0.419±0.044 nM; HepG2 EC_50_:7720±1370, nM) and puromycin (PfLuc EC_50_:32.7±29.7 nM; HepG2 EC_50_:254±36.6 nM). Of the 47 compounds examined, 15 were found to be active against liver-stage parasites (EC_50_ values <1 μM (10–880 nM)), nine of which demonstrated a minimal fivefold difference between parasite activity and hepatocyte toxicity (Supplementary Fig. 1). Interestingly, six compounds were more potent in the liver stage than in the asexual stage. The most significant potency change was seen with MMV019066, a propanamide, which had a reduction in EC_50_ from 1.67 μM to 0.31 μM and previously demonstrated cross-resistance with cytochrome *bc*_*1*_ alleles. This was expected given that cytochrome *bc*_*1*_ inhibitors are highly active against liver stages. MMV024038, a quinoline sulfonamide, also demonstrated a reduction in EC_50_ from 228 nM to 10 nM. This compound, however, exhibited some hepatocyte toxicity (146 nM); thus the indirect effects of the host-cell environment on parasite growth could not be ruled out.

The second assay was a late-stage (stage V) gametocyte survival assay, which predicts transmission-blocking activity. Assays were executed in duplicate using a 12-point EC_50_ curve at starting concentrations of 1.25 μM or 12.5 μM (Supplementary Table 1). Positive and negative control compounds included puromycin (0.61±0.11 μM) and atovaquone (> 12.50 μM), respectively. For the late-stage gametocyte assay, 12 compounds resulted in EC_50_ values <1 μM (260–990 nM), 50% of which overlap with the 15 compounds found to have activity in the liver-stage assay. Of the six compounds with complete multi-stage activity, 83% (5/6) demonstrated hepatocyte toxicity >10 × the liver stage EC_50_. Unlike the liver stage, none of the compounds had increased potency against sexual stage compared with the asexual parasites, which was expected as previous literature has found that most currently used antimalarials yield higher EC_50_ values against late-stage gametocytes when compared with the asexual stage EC_50_ (ref. 13). Five compounds, however, demonstrated late-stage gametocyte EC_50_ values within a twofold range of the asexual EC_50_ value.

### Rate of killing assays

**Figure 3:**
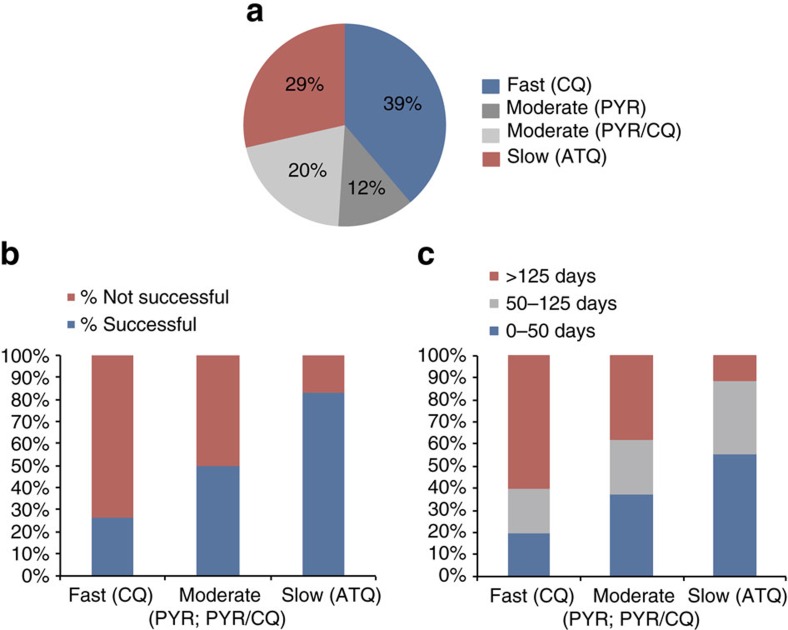
Killing rate trends. (**a**) Proportion of fast (CQ), moderate (PYR or PYR/CQ), and slow (ATQ) compound killing rates in our compound set (49 compounds in analysis). (**b**) Proportion of failed and successful compound selections sorted by killing rate (47 compounds in analysis). (**c**) Successful selections further proportioned out based on amount of time required to result in resistant parasites. Trend in selection success and killing rate was found to be statistically significant (*P*=0.0022) by a one-tailed Fisher's exact test.

To identify whether selection success could be predicted by compound speed of action, and to gain a greater insight into the potential mechanisms of action, assays were performed to test the killing rate (Supplementary Data 1)^33^. To quantify the killing rate, viability time-course profiles for each compound were compared with antimalarials known to have fast (chloroquine), moderate (pyrimethamine) or slow (atovaquone) rate of action. These rates were then compared with other compound characteristics, including cross-resistance profile, selection success, potency and structural characteristics to identify any possible trends. Overall, the compound set resulted in a fairly even distribution of fast, moderate and slow speeds (Fig. 3a), implying there was no bias in the phenotypic screens for a particular killing rate. One compound's rate of killing, MMV666080, was unidentifiable due to compound availability. No correlation was seen between compound structure and speed of action. Additionally, there was no correlation found between killing rate and compound potency against 3D7, nor between potency and selection success (Supplementary Fig. 2).

Comparing the selection success rate to compound rate of killing, we saw a significant positive correlation (*P*=0.0022) between speeds of action and selection success, as determined by a one-tailed Fisher's exact test. (Fig. 3b,c). Of the 12 selections with compounds demonstrating a slow speed of action, 83% (10/12) were successful. This was in contrast to compounds with fast-killing rates of which only 26% (5/19) were successful. Additionally, within each set of successful selections, we noticed a trend within the length of time to generate resistant parasites. For the fast-acting compounds, 3/5 successful selections took >125 days, whereas 5/10 resistant parasites were successfully selected for within 50 days for slow-acting compounds. Therefore, slower compounds typically had greater success and required shorter periods of time to develop resistance when compared with fast-acting compounds. This trend remained relatively consistent within individual labs. These results confirm previous findings of faster acting compounds having a lower propensity for developing *de novo* resistance^34^. Fast-acting compounds are already desirable in the clinic due to a quick clearance of parasites and alleviation of symptoms, as well as their propensity towards slower drug resistance development. The inability to develop resistance *in vitro*, however, even when using slow ramp-up selection methods may imply that these benefits come from the target itself. These genes may have minimal mutational flexibility or inhibitors may target several genes, making them ideal for exploitation in antimalarial development. Alternatively, some compounds may have human host targets instead of parasite targets and affect the erythrocyte directly. Further work is required to distinguish between these possibilities.

## Discussion

Every therapeutic, whether for the treatment of infectious agents or malignant tumours, is in a race against time; a race against the inevitable development of drug resistance leading to reduced clinical effectiveness. Many factors play a role in the development of resistance, from evolutionary and biochemical constraints of the targeted organism to therapy regimens and the overuse or misuse of therapeutics. Not all therapeutics, however, are created equal. A detailed understanding of a targeted organism's biology and the critical factors required to minimize the emergence of resistance provide opportunities to design more effective and long-lasting drugs and drug combinations. This requires an extensive knowledge of the organism, as well as a comprehensive understanding of how resistance evolves.

This study represents the first systematic analysis of cross-resistance in malaria parasites. We have assessed 50 antimalarial compounds with diverse chemical structures, rates of parasite killing and stage specificity. With few exceptions, the compounds studied did not demonstrate significant cross-resistance to previously identified targets, indicating a large potential to identify additional druggable pathways in the parasite and further our arsenal of antimalarial therapeutics. A lack of cross-resistance to known drug targets does not mean that resistance will not quickly develop, however, and the resistance ‘life expectancy' and resulting fitness costs need to be considered for any potential clinical candidate. In selecting for resistant mutants, we found that fast-acting compounds are harder to develop resistance against and generally have a longer onset of resistance when compared with slow-acting compounds. Given that compound killing rates are thought to be determined primarily by their mode-of-action, this resistance feature may be largely due the target or targets themselves. Moving forward, it will be important to verify if this trend holds in compounds with multi-stage activity, as only a small fraction of our compounds (6/50) demonstrated activity against asexual, sexual and liver stage parasites, and multi-stage active antimalarials will be vital in achieving malarial elimination. In addition to killing rate, our compound set provides a list of various physicochemical and structural features that may additionally be predictive of selection success, and the addition of our set to future screens may provide an eventual predictive model, focusing on compounds less likely to be prone to resistance development (Supplementary Figs 3 and 4). Fast-acting compounds have already been a focus in therapeutic development as they rapidly stop disease progression and avoid severe complications, but the additional benefit of reduced evolution of resistance makes them even more attractive candidates for future antimalarial designs.

## Methods

### Compound origin and computational clustering

Compounds were all publically available and obtained from a variety of sources, including the medicines for malaria venture (MMV) malaria box^10^, the GlaxoSmithKline Tres Cantos Antimalarial Set^11^, the University of Dundee and the Broad Institute's Diversity Oriented Synthesis libraries, as indicated in Supplementary Data 1. Compound similarity was calculated using the flexible MCS (FMCS) finder package in R. Clustering was conducted using hclust with a ward.D2 method setting. Pairwise distances were calculated between Tanimoto coefficient values from fmcsR.

### Strain culture origins and propagation

*P. falciparum* parent strains 3D7 and Dd2 used for selection were obtained from the labs of Dan Goldberg and David Fidock, respectively. The Fidock lab additionally supplied the Dd2Δexo strain for selections with MMV000787 and MMV023367. Functional assay and cross-resistant strains were obtained from the labs of Elizabeth Winzeler, Dyann Wirth, David Fidock and GlaxoSmithKline, as listed in Table 1.

Asexual parasites were grown with 5% haematocrit at 37 °C in RPMI (Rosewell Park Memorial Institute)-1640 medium supplemented with 0.014 mg ml^−1^ hypoxanthine, 38.4 mM HEPES, 0.2% NaHCO_3_, 0.2% glucose, 3.4 mM NaOH, 0.3 g l^−1^ glutamine and 5% AlbuMAX II. Depending on lab preferences, parasites were grown either in the presence or absence of 0.05 mg ml^−1^ gentamicin and 4.2% human O^+^ serum (Interstate Blood Bank, Inc.). Cultures were maintained in a gas mixture of 5% O_2_, 5% CO_2_ and 90% N_2_. When not undergoing selections, cultures were maintained with media changes every other day, keeping parasitemia values at 0.3–4%.

Frozen stocks were prepared using one of two methods: (1) by freezing 100% red blood cells (RBCs) at ∼5% parasitemia with equal volumes of a freezing solution composed of 28% glycerol, 3% sorbitol and 0.65% NaCl; (2) freezing 0.2 ml RBCs at ∼5% parasitemia with 0.3 ml serum and 0.5 ml of a glycerol solution. Stocks were thawed out by slowly adding 1/5 volume of 12% NaCl, followed by five volumes of 1.6% NaCl. Parasites were then spun down at 800*g* for 5 min at room temperature and washed with supplemented media before standard culturing methods.

### Cross-resistance and functional assays

EC_50_ assays were conducted using either a 48 or 72-h assay based on lab preference and specialty (internal assay variance summarized in Supplementary Fig. 5). The 48-h assay was carried out in a 96-well format following standard methods using the ^3^H-hypoxanthine incorporation assay^35^. The raw EC50 values corresponding to this assay can be found in Supplementary Data 3. Briefly, this assay relies on the parasite incorporation of labelled hypoxanthine that is proportional to *P. falciparum* growth. A culture of parasitized RBCs (0.5% parasitemia with a percentage of ring stage higher than 70% of total parasitemia, 2.0% haematocrit) in RPMI-1640, 5% AlbuMAX and 5 μM hypoxanthine was exposed to drug serial dilutions. Plates were incubated for 24 h at 37 °C, 5% CO_2_, 5% O_2_ and 90% N_2_. After 24 h of incubation, ^3^H-hypoxanthine was added and plates were incubated for an additional 24-h period. After that, parasites were harvested on a glass fibre filter using a TOMTEC Cell Harvester 96. Filters were dried and melt-on scintillator sheets were used to determine the incorporation of ^3^H-hypoxanthine. Radioactivity was measured using a microbeta counter. Data were normalized using the incorporation of the positive (parasitized RBCs without drug) and negative (same culture with artesunate at 2 μM) controls. All assays were conducted in triplicate using three independently grown cultures, and EC_50_ values were determined using the XLfit add-in module for Excel (version 2.3.1) from Grafit (version 5.3.1.3), where parameter *h* is the assay hill slope, *x* is the compound concentration and *y* is the inhibition of growth effect. The XLfit equation is:


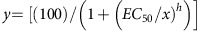


Additionally, any EC_50_ outliers were removed using a single iteration of Grubbs' test, eliminating values with *z*-scores above the critical *z-*value (*α*=0.05).

Alternatively, a SYBR Green-based proliferation approach was used for the 72-h assay, as previously described^32^. Briefly, a culture of either synchronized or asynchronous parasitized RBCs (0.3% parasitemia and 4% haematocrit) in screening medium (identical to supplemented medium above except no serum was added) was exposed to serial drug dilutions. Plates were incubated at 37 °C and gassed with 93% N2, 4% CO2 and 3% O2 for 72 h. Following incubation, 10 × SYBR Green I (Invitrogen; supplied in 10,000 × concentration) in lysis buffer (20 nM Tris-HCL, 5 mM EDTA, 0.16% Saponin wt per vol and 1.6% Triton X vol per vol) was added to the wells and the plates were incubated overnight at room temperature before plate reading. EC_50_ assays were repeated three times, taking the average EC_50_ value from each assay set, and removing any outliers using a single iteration of Grubbs' test, eliminating values with *z*-scores above the critical *z*-value (*α*=0.05). The raw EC50 values for synchronized parasites assayed in a 384-well format are listed in Supplementary Data 4. The raw EC50 values for asynchronous parasites assayed in a 96 or 1536-well format are listed in Supplementary Data 5.

EC_50_ values for each strain were compared with those of the corresponding parent strain to determine fold-shift changes and identify resistance. To minimize the cause variance between assays, all comparisons were conducted between strains assayed under identical conditions. A one-way ANOVA followed by a Dunnet's post-test was conducted in GraphPad Prism to identify significant changes between the parent and resistant strain. For the purposes of this study and to minimize false positive, compounds displaying >5-fold EC_50_ shifts and determined to be significantly different from its corresponding parent by a one-way ANOVA analysis were identified as having potentially non-novel targets.

### Cross-resistance computational analysis

Computational analysis of cross-resistance was conducted in R. Briefly, EC_50_ fold shift ratios were calculated using log transformed EC_50_ values from the parental and resistant strains. As EC_50_ assays were run across multiple labs, any strain fold-shift calculations were conducted between strains run within the same lab to minimize error due to assay variability. The natural log of the ratios was loaded into R, and a heatmap analysis was executed using heatmap.2 from the gplots package. Compounds were clustered by structural similarity (fmcsR) and strains were clustered by column mean values.

### Evolution of compound-resistant lines

Based on the compound speed of action and lab specialty, selections were conducted using a high-pressure intermittent selection method, a step-wise selection method, or a constant selection method. For high-pressure selections, ∼1–2 × 10^9^ parasites were treated at a concentration of 3–10 × EC_50_ until parasites could not be seen by microscopy (4–10 days). Following treatment, compound pressure was removed and cultures were allowed to recover. Once healthy parasites were seen and parasitemia reached ∼2%, compound pressure was reinstated. For step-wise selections, ∼1 × 10^8^ parasites were treated at a starting concentration resulting in a reduced growth rate of 50%. Cultures were examined daily by microscopy, increasing compound concentration in increments of 5–10% as needed to maintain a 50% reduced growth rate. Selections were carried out until cultures achieved a reproducible EC_50_ fold shift of >3 × . Finally, constant selections were conducted in a similar manner to the high-pressure intermittent method with the exception that compound pressure was never removed. Following successful selection, cultures were cloned out using a limiting dilution method as previously described^36^. Selections were terminated after 200 days if resistance could not be obtained.

### Library preparation and analysis of sequenced samples

gDNA was obtained from parasites by washing infected RBCs with 0.05% saponin and isolating the gDNA using a DNeasy Blood and Tissue Kit (Qiagen), following the standard protocols. To prepare the sequencing libraries, gDNA was tagmented and amplified with the Nextera XT kit (Cat. No FC-131–1024, Illumina) using the standard dual index protocol, and sequenced on the Illumina HiSeq 2500 with a RapidRun mode, sequencing 100 base pairs deep on either end of the fragments. Following sequencing, reads were aligned to the *P. falciparum* 3D7 reference genome (PlasmoDB v. 13.0), following the Platypus pipeline as previously described, with the exception that SNVs and INDELs were called with GATK's HaplotypeCaller^37^. To identify valid variants, mutations were filtered using general recommendations from GATK (Supplementary Table 2). Following the initial filtration, mutations where read coverage were <5 and/or where mixed read ratios were>0.2 (reference/total reads) across all samples were removed.

### Rate of killing and multi-stage activity assays

Rate of killing was determined for each compound following a previously described methodology, which uses the invasion of fresh erythrocytes as surrogate of parasite viability^33^. Briefly, parasites were treated with compounds for 48 h. Compound was washed out and fresh-labelled erythrocytes added to the treated parasites. Double stained erythrocytes (RBC label plus parasite DNA label) were quantified and per cent of survival determined.

Liver-stage activity was determined by pretreating hepatic human cells (HepG2) for 2 h with compound in 1,536-well plates infected with freshly dissected *P. berghei* sporozoites. After 48 h of incubation, the viability of *P. berghei* exoerythrocytic forms was measured by luminescence reaction light output using BrightGlo (Promega). Varying levels of compound concentration were used (5 μM or 50 μM) due to the stock concentration supplied.

To test sexual-stage activity, compounds were tested against late-stage gametocytes using a MitoTracker fluorescent-based assay^13^. Specifically, synchronized stage V gametocytes were incubated with compound for 72 h. MitoTracker Red CMXRos (Life Technologies) was added to each well (final concentration: 500 nM) together with saponin to lyse the RBCs. Each plate was then imaged with an Operetta High Content Imaging System (Perkin Elmer) for fluorescence (590–640 nm). Varying levels of compound concentration were used (1.25 μM or 12.5 μM) due to the stock concentration supplied.

The gametocytocidal activity was measured using high-content image analysis software (Harmony, Perkin Elmer). The readout was based on number of metabolically active gametocytes per well.

### Cheminformatics predictors

We evaluated the association between acquisition of drug resistance and pharmacological, physicochemical and structural characteristics of the 48 compounds for which selection was attempted. Specifically, we considered the following properties: (1) pharmacological properties measured in this study: killing rate, toxicity and EC_50_ at three different stages of parasite development; (2) 51 physicochemical descriptors obtained with QikProp software^38^ and 129 descriptors obtained with VolSurf+ (ref. 39); and (3) 2,694 extended-connectivity fingerprints that encode circular substructures with a bond diameter of 10, generated by ChemAxon software (ChemAxon, Kft.)^40^; and (4) 194 hierarchical scaffolds associated with the compounds generated by HierS software^41^.

For each feature, we measured statistical significance of its relationship to the selection success, taking into account the nature of the features^42^. In particular, structural features (that is, extended-connectivity fingerprints and scaffolds) are binary and indicate the presence or absence of each structural element in a compound. For these reasons, hypergeometric statistical tests were applied to structural features and *t*-tests were applied to pharmacological and physiochemical features to measure statistical significance.

### Data availability

All 66 resistant *P. falciparum* lines will be deposited to the Malaria Research and Reference Reagent Resource Center (MR4) and will be made available on request to E.A.W. All relevant sequence data have been deposited in the National Center for Biotechnology Information (NCBI) Sequence Read Archive database with accession code SRP069308. The authors declare that all other data supporting the findings of this study are available within the article and its Supplementary Information files.

## Additional information

**Accession codes**: All relevant sequence data have been deposited in the National Center for Biotechnology Information (NCBI) Sequence Read Archive database with accession code SRP069308.

**How to cite this article**: Corey, V. C. *et al.* A broad analysis of resistance development in the malaria parasite. *Nat. Commun.* 7:11901 doi: 10.1038/ncomms11901 (2016).

## Supplementary Material

Supplementary Information Supplementary Figures 1-5 and Supplementary Tables 1-2

Supplementary Data 1 Summary of killing rate and selection success for compound panel. Compounds rate of killing profiles were determined based on a comparison analysis to fast (chloroquine - CQ), moderate (pyrimethamine - PYR), and slow (atovaquone - ATQ) compounds profiles. Time length to obtain mutants was analyzed, with 0-50 days, 50-125 days, and > 125 days corresponding to short, moderate, and long, respectively. Selection success was quantified by the generation of resistant mutations with an EC50 fold shift {greater than or equal to} 2. Compounds with successful selections are highlighted in green. Two compounds are noted as having NA selection success, as they were removed from the study due to cross-resistance with a strain generated in this study (MMV028895) or compound instability (MMV665824).

Supplementary Data 2 Whole genome sequencing summary for MMV008149.

Supplementary Data 3 Raw EC50 and Hill-Slope Data for strains analyzed in a hypoxanthine 48-hour assay with synchronized parasites in a 96-well format.

Supplementary Data 4 Raw EC50 and Hill-Slope Data for strains analyzed in a SYBR Green 72-hour assay with synchronized parasites in a 384-well format.

Supplementary Data 5 Raw EC50 and Hill-slop data for strains analyzed in a SYBR green 72-hour assay with asynchronous parasites in a 96- or 1536-well format.

## Figures and Tables

**Table 1:** Summary of mutated strains testing cross-resistance

**Strain**	**Target/pathway**	**Parent strain**	**SNVs**	**References**
ATQ-R4	cytb	3D7	M133I and L144S	15
ATQ-R5	cytb	3D7	F267V	15
CYTb-G131S	cytb	Dd2	G131S	16
CYTbQi	cytb	Dd2	G33A	17
GNF156R	PfCARL	Dd2	L830V, S1076I and M81I	14
NITD609-R2	PfATP4	Dd2	T418N and P990R	18
GNF449-R2	PfATP4	Dd2	I203L and P990R	19
PfATP4-Mut2	PfATP4	W2	P412L	20
PfATP4-Mut1	PfATP4	W2	V178I	Unpublished
PfATP4-Mut3	PfATP4	3D7	F917L	20
TM90C2A	DHFR (folate pathway)	3D7*	Unknown (MR4 origin)	28
PfCRT-Q352R	PfCRT	Dd2	Q352R	29
DHODH	DHODH	3D7	E182D	30
hsp90	cellular chaperone	Dd2	D88Y	Unpublished
ProRS	prolyl t-RNA synthetase	Dd2	L482H	31

^*^For TM90C2A, there was no official parent strain, so the clone was compared with 3D7 sensitive strain for cross-resistance.

Fifteen mutated strains and their respective parents were used to test for potential cross-resistance present in our compound panel. Mutations (SNVs) responsible for resistance are listed for each strain, along with their corresponding parents and origin.

**Table 2:** SNV and INDEL mutations in MMV008149

	**MMV008149-F1-ClB2**	**MMV008149-F1-ClB7**	**MMV008149-F2-ClE2**	**MMV008149-F2-ClE3**	**MMV008149-F3-ClB1**	**MMV008149-F3-ClC10**
Genome coverage (*x*)	84.13	81.56	74.81	90.93	82.73	72.93
% Covered by 15 or more reads	95.6	95.7	95.6	95.7	95.8	95
**SNVs identified**						
Total mutations	3	3	5	1	4	4
Intergenic	0	2	4	0	1	3
Intronic	0	0	0	0	0	0
Synonymous	2	0	0	0	2	0
Non-synonymous	1	1	1	1	1	1
Genes mutated in all samples (mutation)			*mal_mito_3 (cytochrome bc1)*			
						
**INDELs identified**						
Total mutations	6	0	1	2	1	1
Intergenic	1	0	1	2	0	1
Intronic	1	0	0	0	0	0
Frame shift	1	0	0	0	1	0
Codon INDEL	3	0	0	0	0	0
Genes mutated in all samples (mutation)			None			

SNV and INDEL mutations were called and filtered using HaplotypeCaller for six clones isolated from three MMV008149 resistant flasks.
