# Supplementary material for: A broad analysis of resistance development in the malaria parasite
Source: Nat Commun. 2016 Jun 15;7:11901. doi: 10.1038/ncomms11901 (PMC4912613; doi:10.1038/ncomms11901)
Supplement: Supplementary Information — Supplementary Figures 1-5 and Supplementary Tables 1-2 [file ncomms11901-s1.pdf]

Supplemental Information

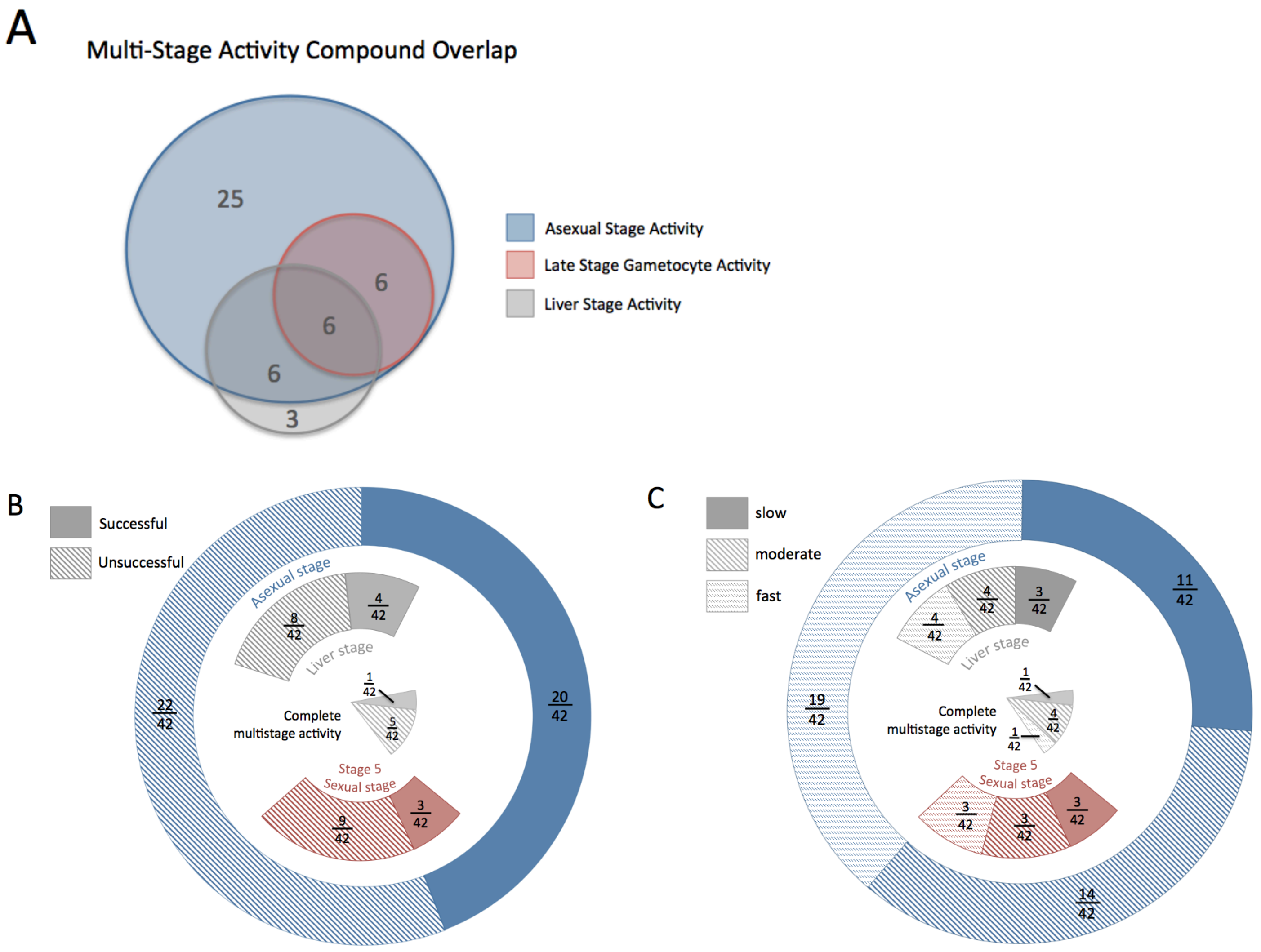

Supplementary Figure 1: Multi-stage activity. (A) Venn diagram summary of multi-stage activity. (B) Breakdown of activity based on selection success. Outer circle represents compounds with asexual activity, first inner circle represents dual stage activity and final pie represents complete multi-stage activity. A potency cut-off of 1µM was required to be considered active against any particular stage. (C) Breakdown of activity based on killing rate. Same pattern as seen in B applies.

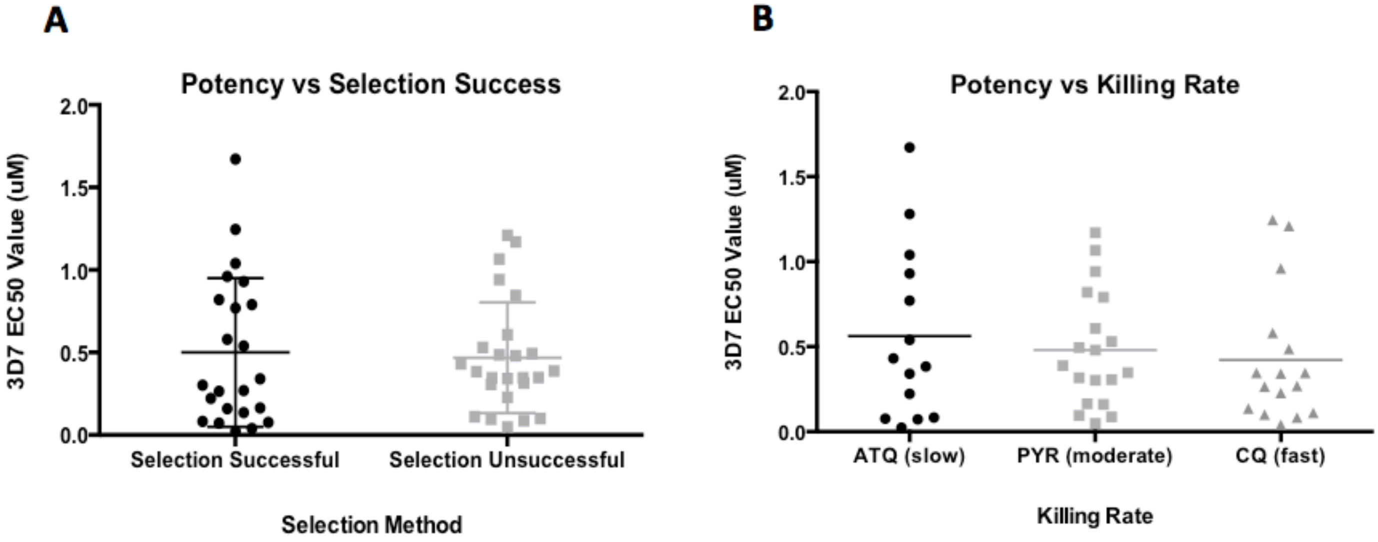

Supplementary Figure 2. Compound potency vs selection success and killing rate. Compound potency against a 3D7 parent strain was compared between groups divided by selection success (A) and killing rate (B) to determine if potency could be a predictor for either group.

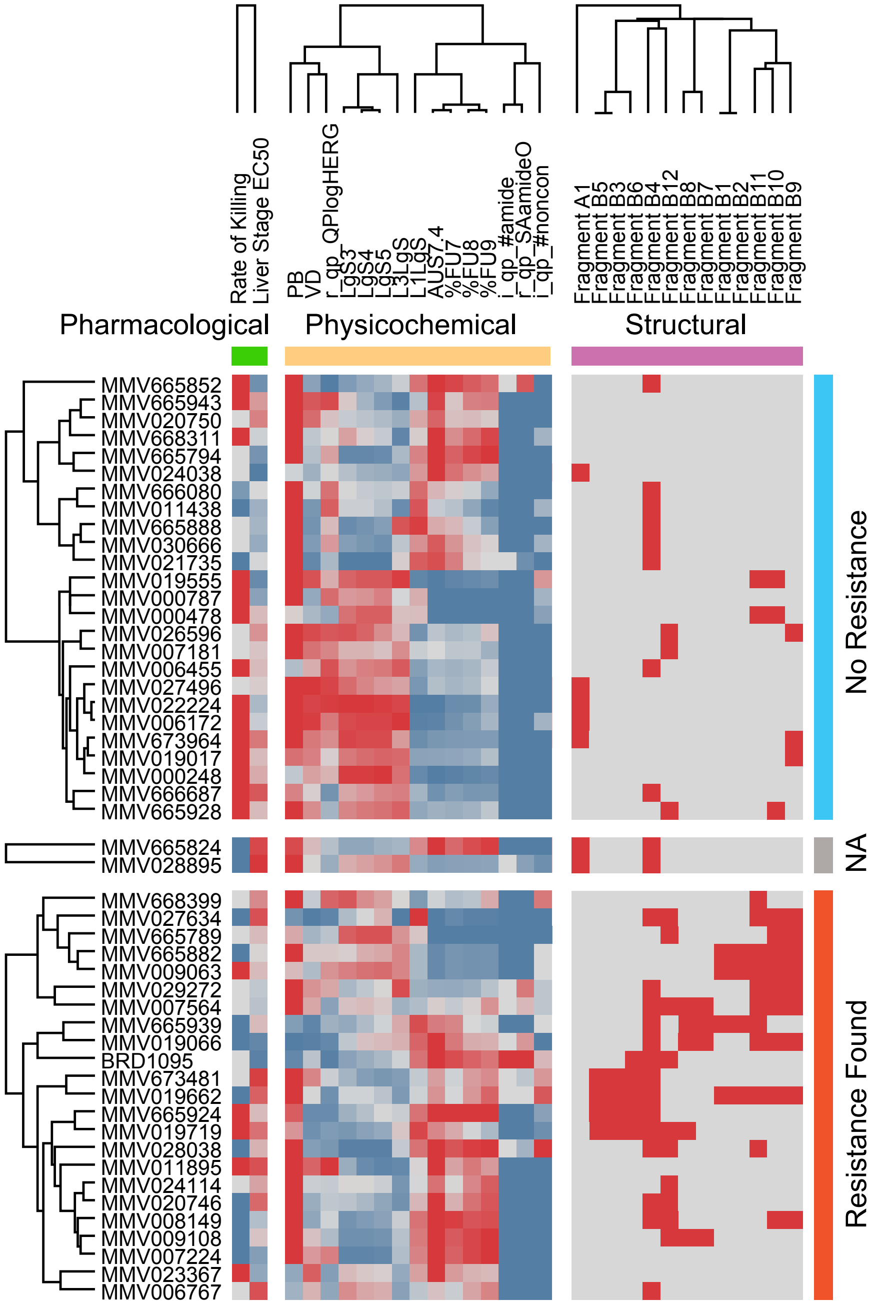

Supplementary Figure 3. Pharmacological and *in silico* features enriched across selection groups. The heat map consists of 48 compounds and a total of 30 statistically significant features ($p<0.05$) (Supplementary Table 1A). The 13 physicochemical features and two pharmacological features were first converted into rank values and then scaled into (-1,1)-interval (red = 1, blue = -1) for the heat map coloring. The presence and absence of structural fragments within corresponding compounds was colored in red and gray, respectively. Each of the three feature groups were independently hierarchically clustered (column trees), where Tanimoto similarity was used for structural features and Pearson correlation was used for pharmacological and physiochemical features. Compounds within each of the two groups (successful and unsuccessful selection) were independently hierarchically clustered using Pearson correlation across their feature profiles.

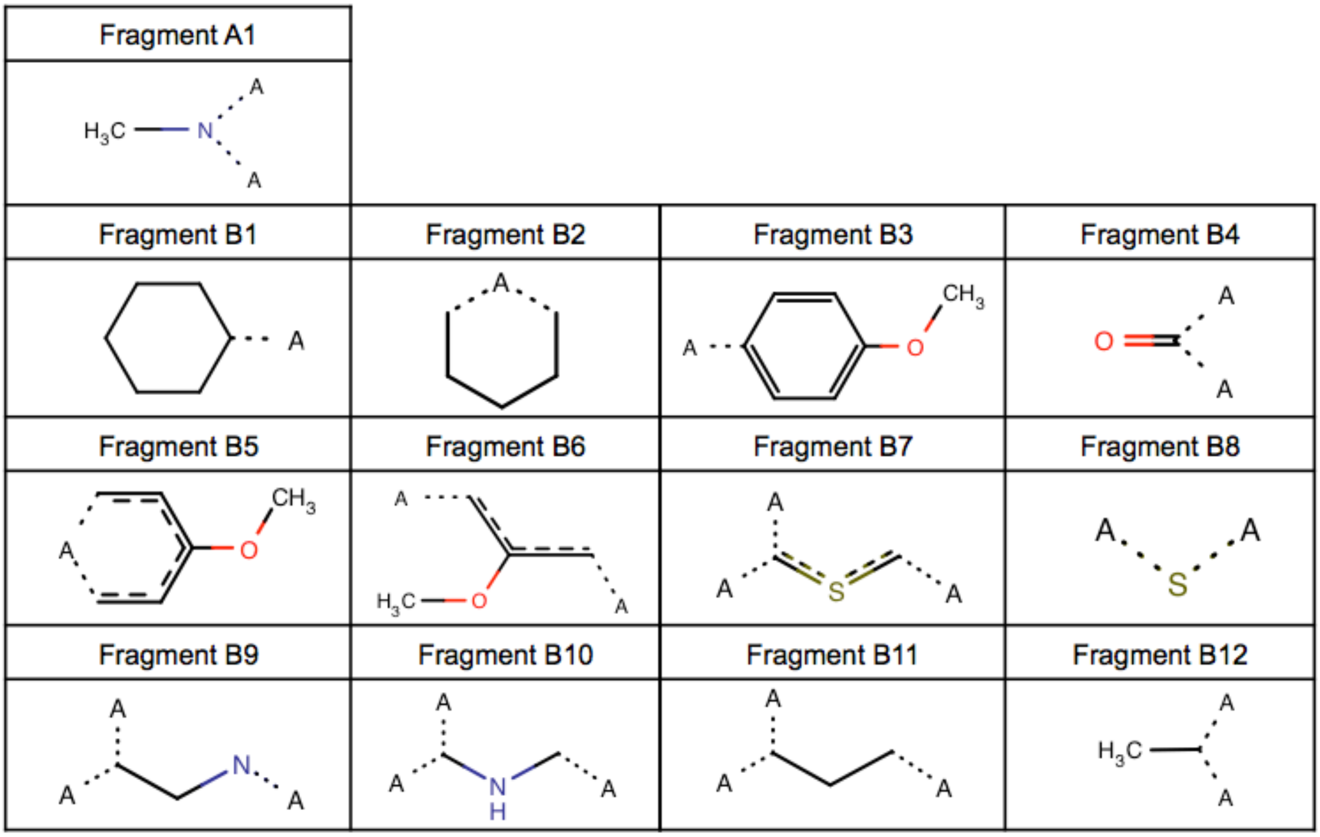

Supplementary Figure 4. Twelve significantly enriched structural fragments. Out of 2888 structural fragments, 13 were found to be significantly enriched within one of the two selection groups as per hypergeometric statistical tests ($p<0.05$). Twelve structural fragments (B1-B12) were enriched in compounds with successful selection, whereas 1 fragment (A1) was enriched in compounds where resistance was not obtained.

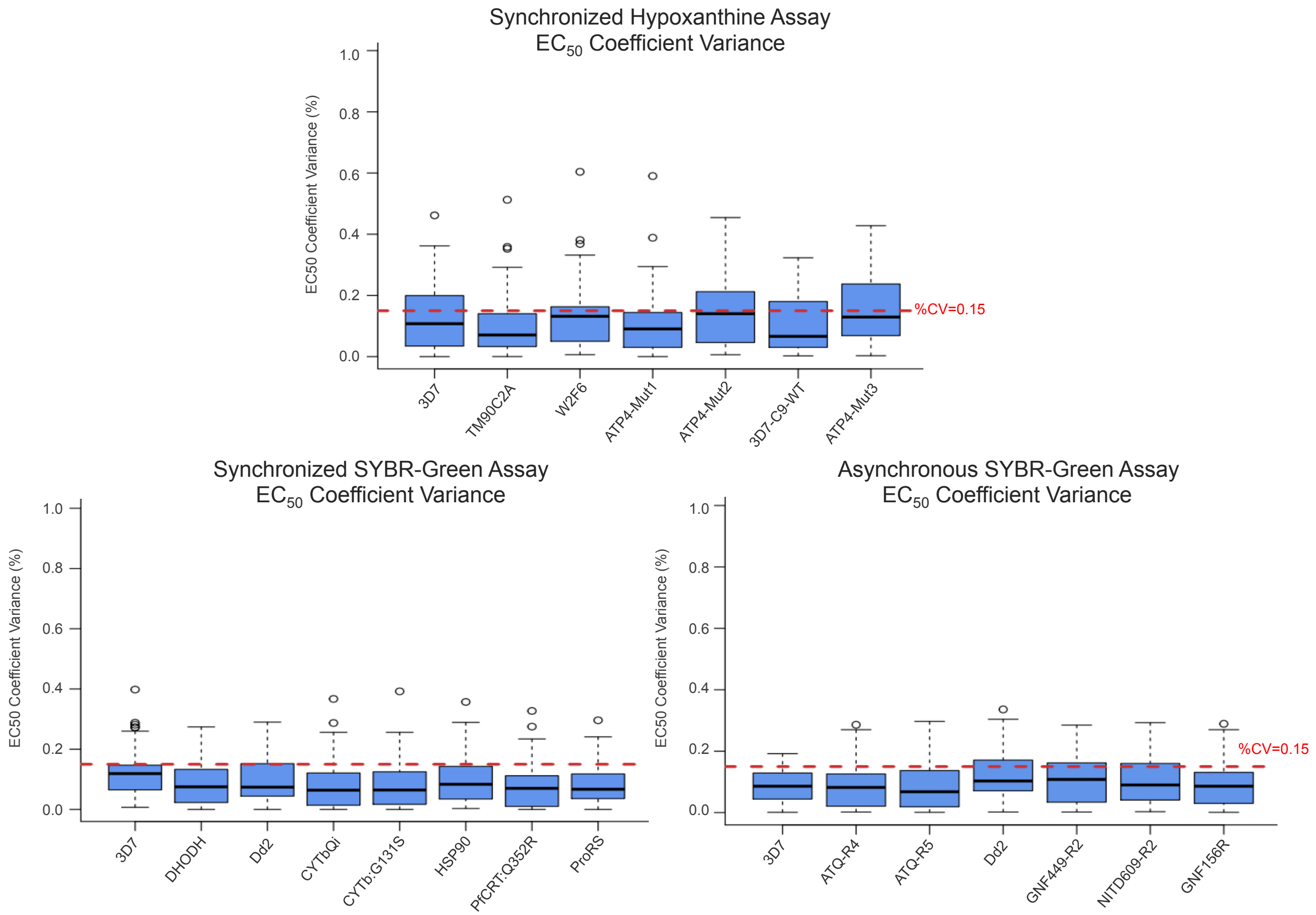

Supplementary Figure 5. EC_{50} Assay Variance. For each of the three assays, boxplots were generated to visualize assay variance. The median is indicated in each box with a black line, and the dotted red line indicates a coefficient variance of 15%.

Compound	3D7 EC_{50} (µM)	Liver Stage EC_{50} (µM)	Hepatocyte Toxicity (µM)	Max Liver Conc. (µM)	Stage 5 Gametocyte EC_{50} (µM)	Max Gametocyte Conc. (µM)	EC_{50} fold shift compared to asexual stage			Liver Stage / Hepatocyte Toxicity
							Liver Stage EC_{50} (µM)	Hepatocyte Toxicity (µM)	Stage 5 Gametocyte EC_{50} (µM)	
MMV000248	0.306	5.552	20.64	50	1.6	12.5	18.14	67.45	5.23	3.72
MMV000478	1.066	>5	>5	5	>1.25	1.25	>5	>5	>1.25	no effect
MMV000787	0.942	0.6001	2.43	50	5.67	12.5	0.64	2.58	6.02	4.05
MMV006172	0.095	2.065	4.554	50	0.919	12.5	21.74	47.94	9.67	2.21
MMV006455	0.53	>5	>5	5	>1.25	1.25	>9.4	>9.4	>2.3	no effect
MMV006767	0.96	>50	>50	50	>12.5	12.5	>52	>52	>13	no effect
MMV007181	0.346	4.786	7.952	50	0.805	12.5	13.83	22.98	2.33	1.66
MMV007224	0.222	0.5144	1.868	50	2.52	12.5	2.32	8.41	11.35	3.63
MMV007564	0.58	1.51	>6.6	33	2.21	8.25	2.60	>57	3.81	>21
MMV008149	0.77	0.8824	>5	5	>1.25	1.25	1.15	>6.5	1.62	>5.6
MMV009063	0.79	>5	>5	5	>1.25	1.25	>6.3	>6.3	>1.5	no effect
MMV009108	0.93	4.786	>5	5	0.921	1.25	5.15	>5.3	0.99	>1
MMV011438	0.431	0.6211	>50	50	0.971	12.5	1.44	>116	2.25	>80
MMV011895	0.16	>50	>50	50	>12.5	12.5	>300	>300	>78	no effect
MMV019017	0.48	>5	>5	5	>1.25	1.25	>10	>10	>2.6	no effect
MMV019066	1.672	0.318	>5	5	>1.25	1.25	0.19	>3	>0.7	>15
MMV019555	0.0873	0.3194	4.811	50	0.451	12.5	3.66	55.11	5.17	15.06
MMV019662	0.34	18.86	>50	50	3.68	12.5	55.47	>147	10.82	>2.65
MMV019719	0.164	10.46	20.33	30	5.52	7.5	63.78	123.96	33.66	1.94
MMV020746	0.076	12.11	31.56	50	3.79	12.5	159.34	415.26	49.87	2.61
MMV020750	0.342	8.16	>5	5	>1.25	1.25	23.86	>15	>3.6	>1
MMV021735	0.383	2.691	>50	50	>11.64	12.5	7.03	>130	>32	>18
MMV022224	0.388	0.8013	2.32	50	2.33	12.5	2.07	5.98	6.01	2.90
MMV023367	0.302	0.6106	4.228	50	6.68	12.5	2.02	14.00	22.12	6.92
MMV024038	0.228	0.01081	0.1461	50	0.317	12.5	0.05	0.64	1.39	13.52
MMV024114	0.265	5.937	9.39	50	>12.5	12.5	22.40	35.43	>47	1.58
MMV026596	0.346	6.474	13.42	50	6.35	12.5	18.71	38.79	18.35	2.07
MMV027496	0.111	3.173	13.49	50	>12.5	12.5	28.59	121.53	>112	4.25
MMV027634	0.023	>50	11.61	50	>12.5	12.5	>2170	504.78	>543	no effect
MMV028038	0.54	5.355	19.24	50	1.18	12.5	9.92	35.63	2.19	3.59
MMV029272	0.136	1.798	5.366	50	2.23	12.5	13.22	39.46	16.40	2.98
MMV030666	0.486	0.6127	10.17	50	0.993	12.5	1.26	20.93	2.04	16.60
MMV665789	1.246	>5	>5	5	>1.25	1.25	>4	>4	>1	no effect
MMV665794	0.1	0.3748	1.374	5	0.836	1.25	3.75	13.74	8.36	3.67

MMV665852	1.17	0.3432	2.594	5	>1.25	1.25	0.29	2.22	>1.5	7.56
MMV665882	0.0727	3.055	13.25	50	0.323	12.5	42.02	182.26	4.44	4.34
MMV665888	1.21	0.6483	4.362	50	2.98	12.5	0.54	3.60	2.46	6.73
MMV665924	0.82	>5	>5	5	>1.25	1.25	>6	>6	>1.5	no effect
MMV665928	0.494	>5	>5	5	>1.25	1.25	>10	>10	>2.5	no effect
MMV665939	1.04	>5	>5	5	>1.25	1.25	>4.8	>4.8	>1.2	no effect
MMV665943	0.316	5.954	14.29	50	0.407	12.5	18.84	45.22	1.29	2.40
MMV666080	0.845	0.4636	1.309	50	7.21	12.5	0.55	1.55	8.53	2.82
MMV666687	0.3477	21.05	31.52	50	4.34	12.5	60.54	90.65	12.48	1.50
MMV668311	0.048	2.251	5.119	50	0.879	12.5	46.90	106.65	18.31	2.27
MMV668399	0.083	7.038	>50	50	>12.5	12.5	84.80	>602	>150	>7
BRD1095	0.04	0.08262	>50	50	0.259	12.5	2.07	>1250	6.48	>605
MMV673964	0.607	8.988	19.83	50	1.13	12.5	14.81	32.67	1.86	2.21

Supplementary Table 1: Multi-stage activity of compound panel. Compounds were screened against 3 stages of *Plasmodium* parasites to test for activity in asexual stage, late sexual stage, and liver stage. *P. falciparum* 3D7 strain was used for asexual stage, Stage 5 gametocytes from *P. falciparum* NF54 was used for sexual stage, and *P. berghei* sporozoites were used for liver stage. To identify compounds with higher potency to liver stage or sexual stage, EC_{50} fold shifts between the asexual stage and either liver stage or late sexual stage were calculated. Finally, toxicity fold shifts were calculated for the liver stage. Compounds were color coded based on being active (EC_{50} < 1μM) against the following stages: (1) asexual only (white); (2) liver only (grey); (3) asexual and sexual (red); (4) asexual and liver (blue); (5)
asexual, sexual, and liver (purple).

Supplementary Table 2: Applied HaplotypeCaller filters

SNV Filters		*INDEL Filters*	
Filter Name	*Filter Value*	*Filter Name*	*Filter Value*
ReadPosRankSum	> 8.0	*ReadPosRankSum*	< -20
	< -8.0	*QUAL*	< 500
QUAL	< 500	*QD*	< 2
QD	< 2	*DP*	< 7
MQRankSum	< -12.5		
DP	< 7		
